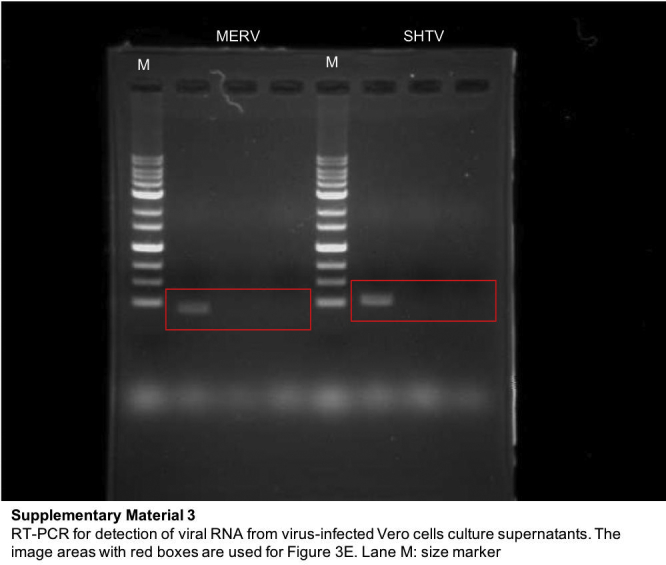# Persistent viruses in mosquito cultured cell line suppress multiplication of flaviviruses

**DOI:** 10.1016/j.heliyon.2018.e00736

**Published:** 2018-08-23

**Authors:** Ryosuke Fujita, Fumihiro Kato, Daisuke Kobayashi, Katsunori Murota, Tomohiko Takasaki, Shigeru Tajima, Chang-Kweng Lim, Masayuki Saijo, Haruhiko Isawa, Kyoko Sawabe

**Affiliations:** a Department of Medical Entomology, National Institute of Infectious Diseases, Tokyo, Japan; b Isotope Imaging Laboratory, Creative Research Institution, Hokkaido University, Sapporo, Japan; c Department of Research Promotion, Japan Agency for Medical Research and Development, Tokyo, Japan; d Department of Virology I, National Institute of Infectious Diseases, Tokyo, Japan; e Department of Agricultural and Environmental Biology, Graduate School of Agricultural and Life Sciences, The University of Tokyo, Japan

**Keywords:** Virology, Molecular biology

## Abstract

In the growth kinetics analysis of flaviviruses in *Aedes albopictus* C6/36 cell lines obtained from the Japanese Collection of Research Bioresources (JCRB) Cell Bank and the European Collection of Authenticated Cell Culture (ECACC), these two cells line showed different viral susceptibility for Zika virus (ZIKV), Dengue virus (DENV), and Japanese encephalitis virus (JEV). Next-generation sequencing (NGS) analysis revealed that the C6/36 JCRB strain was persistently infected with two viruses without showing any cytopathic effects. The complete sequence analysis demonstrated that the one virus was Menghai rhabdovirus (MERV), which has been found from *Aedes albopictus* mosquito. The other virus was a novel virus, designated as Shinobi tetravirus (SHTV). Interestingly, the viral susceptibility of these two strains was almost even for Sindbis virus and Getah virus. We cloned SHTV and MERV from JCRB C6/36 cell line and then re-infected them into another C6/36 cell line, resulting in the reproduction of persistent infection with each virus. ZIKV growth was suppressed in SHTV and/or MERV re-infected C6/36 cells also. To our knowledge, this is the first demonstration that persistent infection with rhabdovirus and/or permutotetravirus suppressed flavivirus replication in mosquito cells.

## 1 Introduction

Arboviruses (arthropod-borne viruses) cause infectious diseases in humans or animals. *Flaviviridae* and *Togaviridae* contain major mosquito-borne viruses such as Zika virus (ZIKV), Dengue virus (DENV), Japanese encephalitis virus (JEV), West Nile virus (WNV), Chikungunya virus (CHIKV), Sindbis virus (SINV), and Getah virus (GETV) [1]. These viruses can replicate not only in humans (or animals) but also in vector mosquitoes. Generally, two *Aedes* mosquitoes (*A. agypti* and *A. albopictus*) are considered to be major vectors for DENV, CHIKV, and ZIKV. To control virus transmission, it is important to understand the behavior of arboviruses in vector mosquitoes.

In dipteran insects (flies and mosquitoes), some anti-viral innate immune systems have been proposed. The innate immunity includes signal cascade pathways (Toll pathway, IMD pathway, and Jak/STAT pathway), RNAi pathways (siRNA pathway and PIWI pathway), and cellular processes (apoptosis and autophagy), though there are some controversial issues (reviewed in [2]). In mosquito virus studies, *A. albopictus*-derived C6/36 cells were primarily cloned and established as viral sensitive cells [3]. Recent studies demonstrated that C6/36 cells have a defect in RNAi pathway due to a mutation in the *Dicer* gene [4, 5]. The defect in the RNAi pathway would contribute to higher susceptibility of C6/36 cells for mosquito-borne viruses [6].

In viral infections, the presence of other parasites or co-infection of viruses is considered to affect viral infectivity. For example, in *Culex* mosquitoes, an infection with WNV suppressed the subsequent infection with a different strain of WNV [7], and an infection with St. Louis encephalitis virus and WNV (both belong to *flaviviruses*) was exclusive to each other [8]. Similarly, Palm Creek virus, an insect-specific *flavivirus*, prevents an infection with WNV [9]. In addition, an intracellular insect bacterium, *Wolbachia*, has been known to influence the ability of *Aedes* mosquitoes to transmit DENV [10, 11, 12, 13, 14].

Recent studies using next-generation sequencing (NGS) technique revealed that mosquitoes and other insects harbored a variety of viruses (or virus-like sequences) in nature [15, 16, 17, 18]. Although the characteristics of most of those viruses except for their genomic sequences have not been identified, the virome is expected to affect arbovirus vector competency of mosquitoes.

In the present study, we identified the viruses that persistently infected to an *A. albopictus* cultured cell line. We could successfully isolate these viruses and assess the arbovirus vector competency of the cells infected with these viruses.

## 2 Materials and methods

### 2.1 Cell culture and viruses

*A. albopictus* cell line C6/36 was purchased from Japanese Collection of Research Bioresources (JCRB) and European Collection of Authenticated Cell Culture (ECACC). C6/36 cells were maintained in Eagle's minimum essential medium (MEM, Sigma) supplemented with 10% fetal bovine serum (FBS) and 2% non-essential amino acids (Sigma) at 28 °C in 5% CO_2_. Mammalian BHK-21 cells and Vero cells were cultured in MEM containing 10% FBS and maintained at 37 °C in 5% CO_2_. Cell growth curves and cell viability were determined by counting cell numbers at each time point using hemocytometer under trypan blue staining.

ZIKV (strain MR766-NIID, GenBank accession no. LC002520), JEV (strain JEV/sw/Mie41/2002, GenBank accession no. AB241119), DENV (strain D1/Huh/Saitama/NIID100/2014, GenBank accession no. LC011945), and SINV (strain NC001547, GenBank accession no. NC001547) were cultured in Vero cells, and GETV (strain 12IH26, accession No.: LC152056) was cultured in BHK-21 cells. Viral titers were determined by plaque assay using Vero cells (ZIKV, JEV, DENV, and SINV) or BHK-21 cells (GETV), and the resultant viruses were subjected to further analysis.

### 2.2 Determination of the complete viral genome sequence

Viral RNAs were recovered from a supernatant of C6/36 JCRB strain cell culture. Briefly, the supernatant was treated with DNase and RNase, and the total RNA was isolated using Isogen II reagent (Nippon Gene). Viral genomic sequences were determined by using Ion PGM System (Thermo Fisher) followed by RACE sequencing as described elsewhere [19]. The complete nucleotide sequences of the viruses reported in the present study have been submitted to the DDBJ/GenBank/EMBL database under accession numbers LC270813 (Shinobi tetravirus: SHTV) and LC270812 (Menghai rhabdovirus strain kunoichi: MERV).

### 2.3 Phylogenetic analysis

Phylogenetic analysis was conducted using sequences for selected viruses in *Rhabdoviridae* or *Permutotetraviridae*, based on the protein sequences deduced from the conserved RdRp gene. A multiple sequence alignment matrix was created using DDBJ ClustalW (http://clustalw.ddbj.nig.ac.jp/) with default settings. The aligned matrix data were confirmed manually, and the amino acid sequences that were completely conserved among viruses (complete deletion of gap/missing data) were analyzed using the maximum-likelihood method. The statistical significance of the resulting tree was evaluated using a bootstrap test with 1,000 replications.

### 2.4 Virus isolation and RT-PCR

SHTV and MERV in C6/36 JCRB strain were isolated by limiting dilution method using C6/36 ECACC strain. Infection with SHTV and MERV was confirmed by RT-PCR using PrimeScript one-step RT-PCR kit (Takara) with specific primer sets [SHTV: 5′-TTCTTAGGAATGAGGCTCATG-3′ and 5′-ACTAGGTCGTTGGGGCTCTC-3′ (position 1,648–1,964) and MERV: 5′-GAGGTGTCGGATAAATTTCTAG-3′ and 5′-TTATCTGATAGGTGCCCCTTC-3′ (position 4,960–5,213)]. The cell culture supernatants with single infection with SHTV or MERV were subjected to a blind passage, and the viral infection was confirmed again by RT-PCR as described above. The C6/36 ECACC strain cells persistently infected with SHTV and/or MERV were maintained individually as mock-infected cells. Real-time PCR analysis was carried out using ThermoScript reverse transcriptase (Thermo Fisher) an KOD-plus ver.2 PCR enzyme (Toyobo) with EvaGreen fluorescence (Biotium).

### 2.5 Analysis of growth kinetics in viruses

For the growth kinetics analysis, a total of 1 × 10^6^ C6/36 cells f were plated in 6-well plates and infected with ZIKV, JEV, and DENV at a multiplicity of infection (MOI) of 0.01 and SINDV and GETV at an MOI of 0.001. Small aliquots of the supernatants were collected at every 12 or 24 hours and the titer was determined by plaque assay using Vero cells cultured in 12-well plates.

### 2.6 Statistical analysis

For the growth kinetic analysis of ZIKV, JEV, DEV, SINDV, and GETV, *t*-test between samples (C6/36 JCRB strain and ECACC strain) was carried out for the viral titer at each time point (Fig. 2). For the growth kinetic analysis of ZIKV in C6/36 ECACC strain cells infected with SHTV and MERV, the multiple comparison of dunnett's methods was adopted (Fig. 4).

## 3 Results

**Fig. 1:**
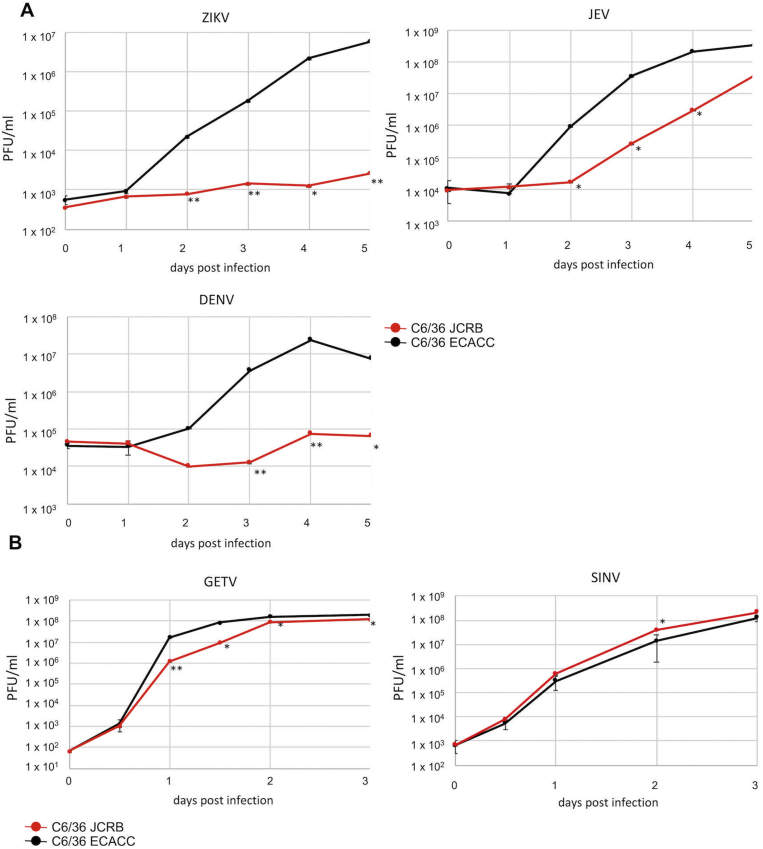
C6/36 cells of JCRB and ECACC strains showed different susceptibility to flavivirus infection. Growth kinetics of arboviruses in C6/36 cell lines. **A**: C6/36 cells of either JCRB or ECACC strain were infected with Zika virus (ZIKV), Japanese encephalitis virus (JEV), or Dengue virus (DENV) at a multiplicity of infection (MOI) of 0.01, and the titers in the supernatants were determined by plaque assay. **B**: C6/36 cells of either JCRB or ECACC strain were infected with Getah virus (GETV) or Sindbis virus (SINV) with MOI = 0.001. Red lines show the titer in C6/36 JCRB strain and black lines show that in C6/36 ECACC strain. The experiment was triplicated, and the standard deviations are also indicated. Asterisks showed the results of *t*-test for the data group at each time points (*p < 0.05, **p < 0.01).

*Aedes albopictus*-derived C6/36 cells are susceptible to a variety of mosquito-borne viruses and well used for arbovirus studies. We also used C6/36 cells for studies of arboviruses but noticed that the viral susceptibility of C6/36 cells was varied among laboratories. Therefore, we purchased several C6/36 strains from different cells banks, JCRB and ECACC, and assessed viral susceptibility using flaviviruses (*Flaviviridae*) and alphaviruses (*Togaviridae*). We determined the growth kinetics of flaviviruses (ZKV, JEV, and DENV) and alphaviruses (SINV and GETV) in C6/36 cells of JCRB strain and ECACC strain. The growth kinetics of flaviviruses in C6/36 JCRB strain was significantly lower than that in C6/36 ECACC strain (Fig. 1A). For example, the titer of ZIKV in the supernatant of C6/36 JCRB strain at 4 dpi was 0.1% of that of ECACC strain. On the other hand, the growth of GETV was just slightly decreased in JCRB strain (Fig. 1B). In the case of SINV infection, no significant differences in growth kinetics were found between JCRB and ECACC strains. Therefore, C6/36 cells of JCRB and ECACC strains had different susceptibility, particularly for flaviviruses.

**Fig. 2:**
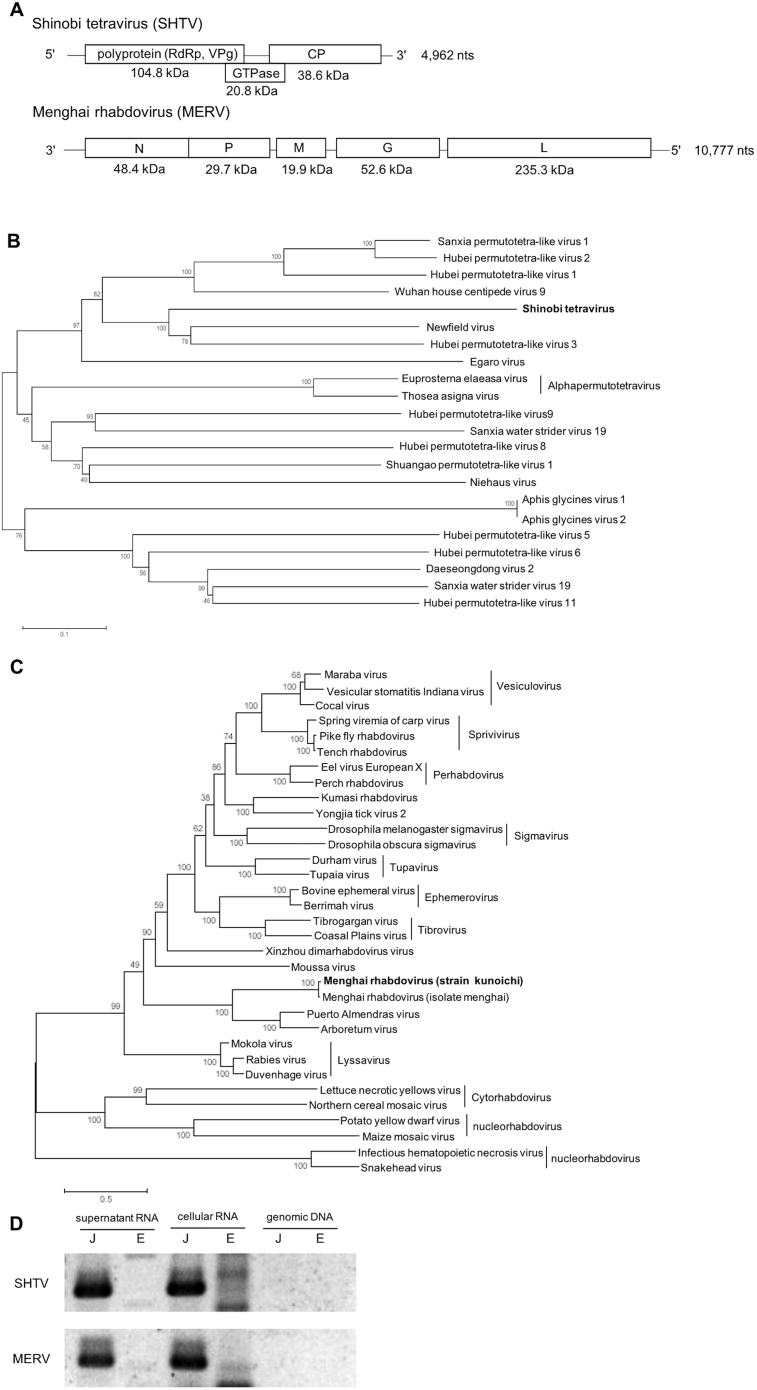
Identification of viruses infected in mosquito cultured cell line C6/36. **A**: Schematics of Shinobi tetravirus (SHTV) and Menghai rhabdovirus (MERV) genomic sequences. Encoded putative genes and their expected sizes are presented (RdRp: RNA-dependent RNA polymerase, VPg: Viral protein genome-linked, CP: capsid protein, N: nucleoprotein, P: phosphoprotein, M: matrix protein, G: glycoprotein, and L: RNA-dependent RNA polymerase). **B**: Phylogenetic analysis of SHTV and other viruses containing *Permutotetraviridae*. The amino acid sequences of putative RdRp were aligned and then analyzed using a maximum-likelihood method. Bootstrap replications are shown on the branches. **C**: Phylogenetic analysis of MERV and other rhabdoviruses. The amino acid sequences of L proteins were analyzed as described. **D**: Detection of SHTV or MERV in C6/36 JCRB strain and ECACC strain. The viral genome was determined by RT-PCR with the template RNA extracted from cell culture supernatant or cultured cells. PCR amplification from genomic DNA extracted from cultured cells were also determined. J: C6/36 JCRB strain, E: C6/36 ECACC strain. The original image is shown in Supplementary Material 1.

In our recent studies, we adopted NGS analysis to detect mosquito-borne viruses [19]. In those experiments, we noticed that the C6/36 cells obtained from the JCRB contained virus-like sequences that would belong to *Rhabdoviridae* or *Permutotetraviridae*-related group. Therefore, we isolated RNA from the culture supernatant of newly purchased C6/36 JCRB strain and determined these virus-like complete sequences with a combination of NGS method and RACE sequencing method. The results indicated that the *Rhabdoviridae* virus was Menghai rhabdovirus (MERV; designated as strain kunoichi), and the other *Permutotetraviridae*-related virus was a novel virus designated as Shinobi tetravirus (SHTV) (Fig. 2A). The strain name “kunoichi” and virus name “Shinobi” means a Japanese covert agent, which is the same meaning with Ninja.

The SHTV genome consists of 4,962 nucleotides (nts) with three open reading frames (ORFs). The blast search indicated that the first ORF (ORF1) encoded 104.8 kDa polyprotein containing RNA-dependent RNA polymerase and viral protein genome-linked (VPg). The second ORF (ORF2) encoded 20.8 kDa protein, and the ORF was located across the first and the third ORFs. The structural prediction by HHpred suggested that the second ORF contained putative GTPase domain. The third ORF (ORF3) encoded putative 38.6 kDa capsid protein (CP).

The MERV genome consists of 10,777 nts with 5 ORFs (N: 48.4 kDa, P: 29.7 kDa, M: 19.9 kDa, G: 52.6 kDa, and L: 235.3 kDa), which is a typical rhabdoviral genomic composition. The comparison of sequences between MERV kunoichi strain (this study) and Menghai strain showed 97% identity in L protein. MERV forms a single cluster with Puerto Almendras virus (41.7% identity with MERV) and Arboretum virus (40.6% identity with MERV) (Fig. 2C). Although the genetic identity of these three viruses was not high, they were found from mosquitoes. It suggests that MERV and these viruses are to be considered as mosquito-specific rhabdoviruses.

In the phylogenetic analysis of SHTV RdRp gene, SHTV had only 33.2% identity with the closest virus, Newfield virus. Even though SHTV showed low similarly with other viruses, SHTV formed a cluster with Newfield virus (isolated from *Drosophila melanogaster*), suggesting that SHTV also comes from dipteran insects including mosquitoes (Fig. 2B). Most of these viruses in this phylogenetic tree were detected in the metagenomic analysis using insect mixes.

Then, to determine whether other C6/36 cell lines also had SHTV or MERV, we performed RT-PCR to detect SHTV and MERV using cultured C6/36 or their culture medium (3 days post-passage) of JCRB or ECACC strain. As the result, neither SHTV nor MERV was detected from ECACC strain and its culture supernatants (Fig. 2D, lanes 1–4). Some non-specific amplifications were appeared in the RT-PCR from the cellular RNA of C6/36 ECACC cells but not from the cell culture supernatant, suggesting that the non-specific bands came from intracellular RNAs. We also carried out NGS analysis using RNA isolated from C6/36 ATCC cell collection strain, but no SHTV- or MERV-related viruses were detected (data not shown). PCR with DNA extracted from JCRB or ECACC strain did not detect any DNA-based amplification for SHTV or MERV (Fig. 2D, lanes 5–6). These results suggested that SHTV and MERV are persistently infecting, not genomic DNA-integrated, viruses unique for C6/36 JCRB strain.

**Fig. 3:**
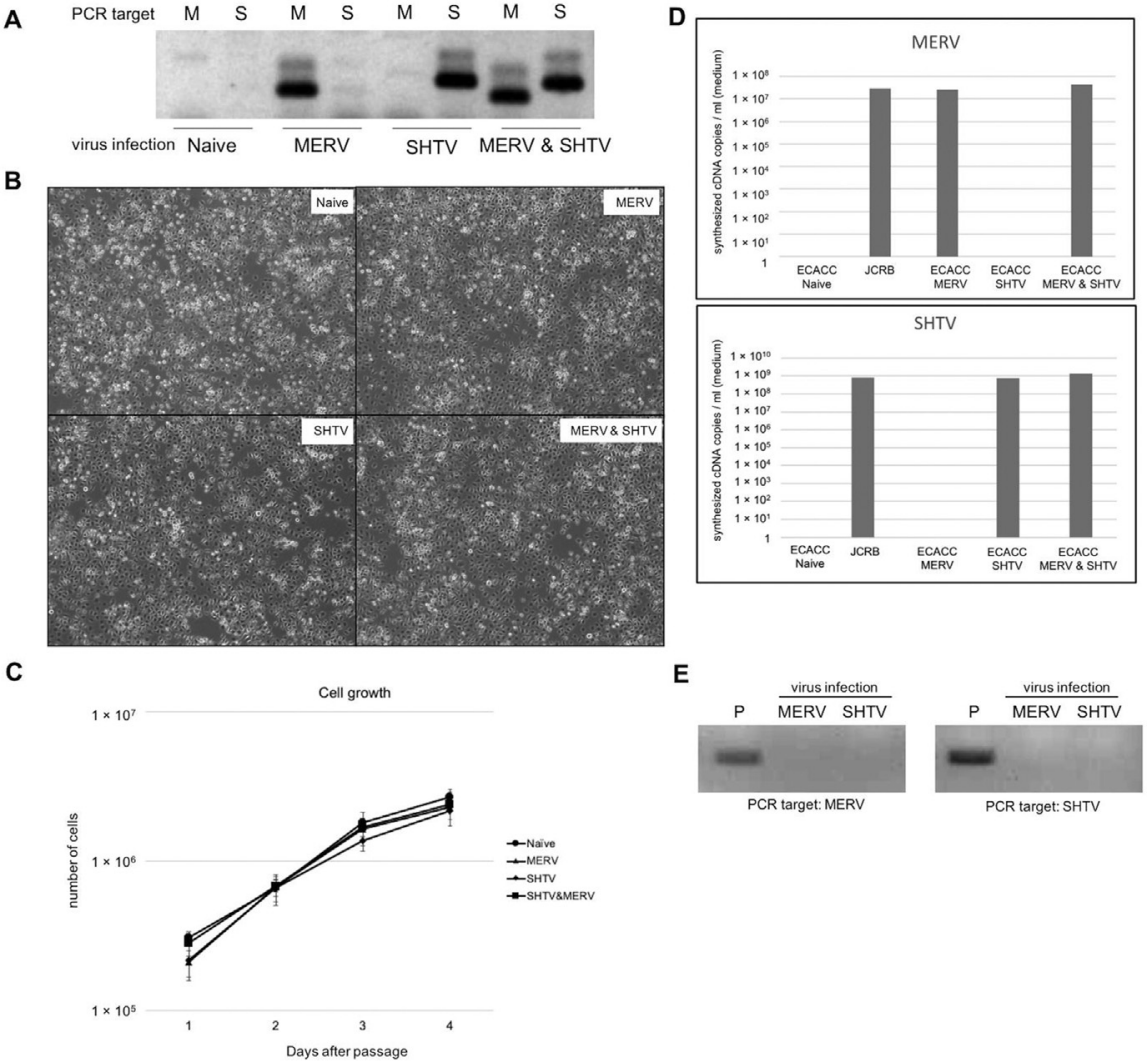
SHTV and/or MERV infection to naïve C6/36 cultured cell line. **A**: Determination of infection of C6/36 ECACC strain with single-isolated Shinobi tetravirus (SHTV) and/or Menghai rhabdovirus (MERV). Viral RNAs in infected cells were determined by RT-PCR (M: PCR targeting MERV, S: PCR targeting SHTV). The original image is shown in Supplementary Material 2. **B**: Morphology of SHTV- and/or MERV-infected C6/36 ECACC strain. Each panel shows the morphology of the cells 6 months (with 29 passages) after infection. **C:** Growth kinetics of MERV and SHTV-infected C6/36 cells of ECACC strain. Error bars showed the standard deviations in the triplicated experiments. **D:** Quantitative RT-PCR (qRT-PCR) for MERV and SHTV in C6/36 cells. Viral RNAs were extracted from cultured supernatant at 2 days after passage. The number of viral genomes was determined in qRT-PCR with PCR-amplified standard curves of MERV or SHTV. **E:** Detection of MERV and SHTV infection in mammalian Vero cells. Virus RNAs in the culture supernatant were determined by RT-PCR at 3 days post infection. P: positive control (RNAs from the supernatant of virus-infected C6/36 ECACC cells). The original image is shown in Supplementary Material 3.

To verify whether the differences in virus susceptibility between C6/36 JCRB and ECACC strains came from the SHTV and/or MERV persistent infection, we first tried to isolate each virus from the culture supernatant of C6/36 JCRB strain by a limiting dilution method. The supernatant of C6/36 JCRB strain cell culture was collected, and then the C6/36 ECACC strain was infected with the dilution series of the supernatant. At 5 days post-infection, the virus in the supernatant was determined by RT-PCR. After the second blind passage, we determined the virus infection again. The single infection with SHTV and MERV was found in the dilution of 2.5 × 10^−6^ and 5 × 10^−6^, respectively. The resultant virus-infected cells were cultured for 15 days with five passages, and the persistent infection with SHTV and/or MERV was determined by RT-PCR (Fig. 3A). We found successful persistent infection with SHTV and/or MERV of C6/36 ECACC strain without changing cellular morphology (Fig. 3B). The growth curves of the cells infected with MERV and SHTV were not different from that of naïve cells. The viability of naïve, MERV-infected, SHTV-infected, and MERV- and SHTV-infected cells were 97.4%, 97.5%, 97.6%, and 96.6%, respectively. Thus the infection of MERV and SHTV did not toxic for C6/36 ECACC cells. We then determined SHTV and MERV titers in C6/36 JCRB strain cells and C6/36 ECACC strain cells infected with SHTV and/or MERV (Fig. 3C). SHTV titers in JCRB strain and SHTV-infected ECACC strain cultured supernatant s were almost even (JCRB: 2.9 × 10^7^ copies/ml, SHTV-infected ECACC: 2.6 × 10^7^ copies/ml). Similarly, MERV titer in MERV-infected ECACC strain (7.9 × 10^8^ copies/ml) was almost even with JCRB strain (8.1 ×10^8^ copies/ml). In the ECACC strain cells infected with both SHTV and MERV, the viral titers of SHTV and MERV were also even to those in JCRB strain cells (SHTV: 4.5 × 10^7^ copies/ml, MERV: 1.4 × 10^9^ copies/ml). These data suggested that viral susceptibility of ECACC strain to SHTV and MERV infection was almost same with that of JCRB strain cells, and SHTV and MERV infection did not affect viral replication each other.

We also tested the infectivity of SHTV and MERV to a mammalian Vero cells, but no viral RNAs were detected in the supernatant of virus-infected Vero cells at 3 days post infection (Fig. 3D). This result suggested that the Vero cells were resistant for SHTV and MERV infection, and SHTV and MERV were considered as insect virus.

**Fig. 4:**
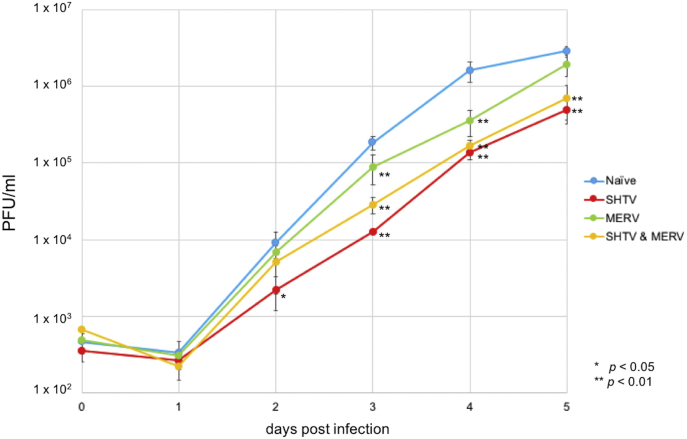
Infection of SHTV and/or MERV suppressed ZIKV growth kinetics in C6/36 cells. Growth kinetics of Zika virus (ZIKV) in C6/36 ECACC strain infected with Shinobi tetravirus (SHTV) and/or Menghai rhabdovirus (MERV). Cells were infected with ZIKV at a multiplicity of infection of 0.01, and the viral titers in the supernatants were determined by plaque assay. Blue: naive C6/36 ECACC strain, Red: SHTV-infected C6/36 ECACC strain, Green: MERV-infected C6/36 ECACC strain, and Yellow: SHTV- and MERV-infected C6/36 ECACC strain.

Using C6/36 ECACC strain cells persistently infected with SHTV and/or MERV, the growth kinetics of ZIKV was determined. The ZIKV growth curve in SHTV- and/or MERV-infected cells delayed, and the titers were relatively lower than those in the naive C6/36 ECACC strain (Fig. 4). For example, the ZIKV titer in SHTV-infected cells was 6.9% at 3 days after ZIKV infection. Thus, the decreases of ZIKV growth kinetics was reproduced in artificially constructed SHTV and/or MERV persistently infected cells, but the suppression was not significant as those in C6/36 JCRB strain.

## 4 Discussion

In this study, we reported that MERV and SHTV persistently infection suppressed flaviviruses replication in the cultured mosquito cell line. The susceptibility of viruses is important for analyses of virological property and highly correlated with succession rate of isolation of the viruses. It indicated that C6/36 cells which are free for MERV and SHTV should be utilized for isolation from specimens. Furthermore, isolated viruses from specimens using MERV and SHTV infected C6/36 cells might have less replicative because of contamination of MERV and SHTV in isolated the viruses. The viruses which have passage history of using MERV and SHTV infected cell should be paid attention to use for several analyses.

The efficiency of arbovirus replication in vector mosquitoes has a large impact on the spread of arbovirus infectious diseases. However, little has been understood about the mechanisms of arbovirus replication in mosquitoes and the mechanisms of anti-viral immunity. It has been proposed that genetic diversity in wild mosquito populations or environments including microbial flora or virome would influence virus susceptibility [20, 21, 22, 23]. As demonstrated in the results of recent metagenomic analyses, insects naturally harbor a variety of viruses regardless of their pathogenicity; however, the function of most of these viruses has not been determined [15, 16, 17, 18]. In the present study, we detected the viruses (MERV and SHTV) persistently infecting *A. albopictus* C6/36 cells. We were able to obtain the C6/36 cells that were not infected with these viruses. Interestingly, the JCRB and ECACC C6/36 cell lines showed significantly different susceptibility, particularly for flaviviruses (Fig. 1), suggesting that the MERV and/or SHTV would contribute to the arbovirus susceptibility. Newly established MERV and/or SHTV persistent infection cell lines also showed the suppressive effect on ZIKV infections. However, the suppression SHTV/MERV-infected C636 ECACC cells were not as significant as that seen in C6/36 JCRB strain even though the SHTV/MERV titers in persistently infected C6/36 ECACC strain cells were almost even to those in C6/36 JCRB strain cells (Fig. 3C). These data suggest that not only MERV and/or SHTV infection but also other factors, such as genetic changes raised during long term cell culture would strongly contribute to the resistance to flavivirus infections. If this idea is true, it suggests that A. albopictus could have a resistance to flavivirus infection upon some genetic mutations. It is also possible that exposure to MERV and SHTV for a longer time induces higher resistance to flavivirus infections because C6/36 JCRB strain would have MERV and SHTV infection for relatively longer periods than that in the MERV- and/or SHTV-infected C6/36 cells prepared in the present study.

In the phylogenetic analysis for rhabdoviruses, MERV appeared to form a cluster with Puerto Almendras virus and Arboretum virus [24, 25]. Because all these viruses have been reported as mosquito-derived viruses, the new cluster would be a group of mosquito-specific rhabdoviruses. This idea was supported by the fact that MERV infection was not detected in mammalian Vero cells (Fig. 3E). MERV Menghai strain was first reported in the virus isolation experiment from *A. albopictus* mosquito using C6/36 cells [24]. This meant that MERV was originated from a wild *A. albopictus* and come into C6/36 cells during the history. The genetic diversity (97% identity of L protein between MERV Menghai strain and kunoichi strain) would support this idea, although we could not neglect another possibility that MERV Menghai stain was found from C6/36 cells that had been persistently infected with MERV like as this work.

In the phylogenetic tree of alphapermutotetraviruses and other related viruses, on the other hand, the relationship between the viruses and their hosts was slightly complicated. The close virus to SHTV was Newfield virus, which was found in dipteran *D. melanogaster* [15]. However, Hubei permutotetra-like virus 3, which was located in the same clade with Newfield virus, was reported as found from a pool of spiders [17]. Thus, the viruses related to SHTV did not form a mosquito virus group simply such as MERV, though the putative hosts of some of the viruses in this phylogenetic tree were mosquitoes (e.g., Daeseongdong virus 2 and Hubei permutotetra-like virus 11) [17,26].

C6/36 cells were known to be first established as highly sensitive cells for DENV and CHIKV infections and to be deficient in RNAi pathway due to a mutation in the *Dicer* gene [3, 4, 5]. In addition to this, no sequence similarity was found between SHTV or MERV and flaviviruses, implying that the ZIKV suppression found in the present study is not likely to be due to the siRNA-mediated pathway or other small RNA-mediated pathways.

In infection of viruses, the presence of other parasites or co-infection of viruses is considered to affect viral infectivity. Examples in *Culex* mosquitoes, an infection of WNV suppressed the subsequent infection of a different strain of WNV [7], and the infection of St. Louis encephalitis virus and WNV (both of them are *flavivirus*) was exclusive each other [8]. Similarly, Palm Creek virus and Nhumirin virus, which were thought to be insect-specific *flaviviruses*, prevent an infection of WNV [9,27]. Thus, some flaviviruses prevent other flavivirus infection in mosquito cells though the detailed mechanisms of these suppressions were not fully understood. More recently, Schultz et al. reported a suppression of ZIKV infection in mosquito cells, which was induced not only by insect-specific flavivirus but also by a *Bunyaviridae* virus, Phasi Charoen like virus [28]. These data and our present study showed that flavivirus infection in mosquitoes could be suppressed by the infection of non-pathogenic, insect-specific viruses which were taxonomically far from flavivirus. Further to say, natural virome in mosquito populations would be important for spread of arbovirus infections. Interestingly, the anti-viral effects appeared to be more specific for flaviviruses than for alphaviruses, suggesting that the suppression would not be simply an induction of cellular anti-viral innate immunity. How the suppression is induced and whether the suppression is functional in adult mosquitoes remains to be elucidated.

## Declarations

### Author contribution statement

Ryosuke Fujita, Fumihiro Kato: Conceived and designed the experiments; Performed the experiments; Analyzed and interpreted the data; Wrote the paper.

Daisuke Kobayashi, Katsunori Murota: Performed the experiments.

Tomohiko Takasaki, Shigeru Tajima, Chang-Kweng Lim, Masayuki Saijo, Haruhiko Isawa, Kyoko Sawabe: Contributed reagents, materials, analysis tools or data.

### Funding statement

This work was supported by the Research Program on Emerging and Re-emerging Infectious Diseases from Japan Agency for Medical Research and Development, AMED, and JSPS KAKENHI 15H04614.

### Competing interest statement

The authors declare no conflict of interest.

### Additional information

Data associated with this study has been deposited at DDBJ/EMBL/Genbank under the accession numbers LC270812 and LC270813.

## Figures and Tables

**Supplementary Material 1:**
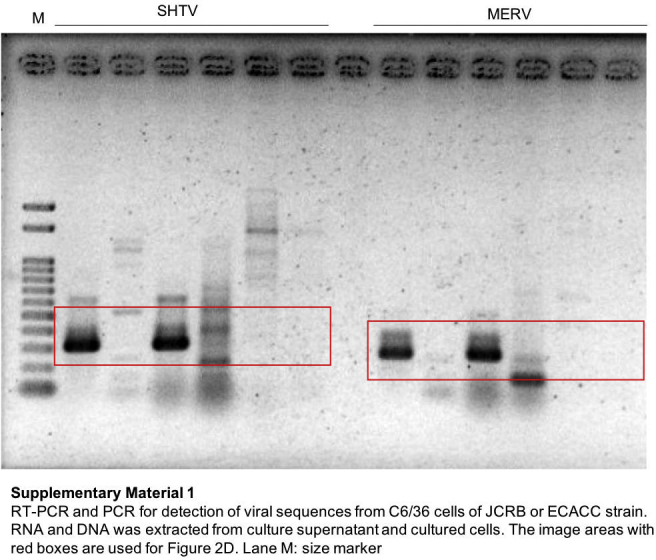


**Supplementary Material 2:**
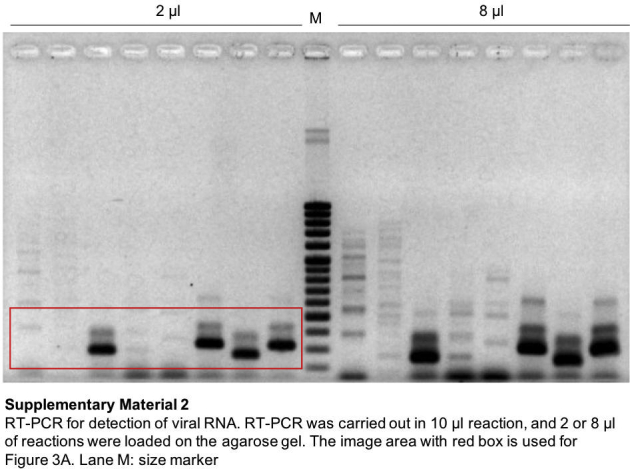


**Supplementary Material 3:**